# Perspective: Thermophoresis and Its Promise for Optical Patterning

**DOI:** 10.1021/acs.langmuir.5c01023

**Published:** 2025-05-13

**Authors:** Ana Jiménez Amaya, Eric H. Hill

**Affiliations:** Institute of Physical Chemistry, 14915 University of Hamburg, Grindelallee 117, 20146 Hamburg, Germany; The Hamburg Center for Ultrafast Imaging (CUI), Luruper Chaussee 149, 22761 Hamburg, Germany

## Abstract

Thermophoresis, the movement of molecules and colloids under a thermal gradient, has been recently shown to be effective for localized trapping, manipulation, and even printing of colloidal particles at interfaces. However, the lack of a broader understanding of the behavior of various molecules and colloidal species poses a challenge to the ongoing development of thermophoresis as a tool for patterning and assembly. In this Perspective, we discuss the thermophoresis of colloids, highlighting the barriers to understanding and predicting these complex systems, as well as recent approaches for measuring and predicting thermophoretic behavior. Further development of thermophoresis-based patterning techniques is crucial to unlocking their potential to advance the field of optically driven colloidal assembly, and critical for the rapid, on-demand fabrication of sensors and devices.

## Graphical abstract

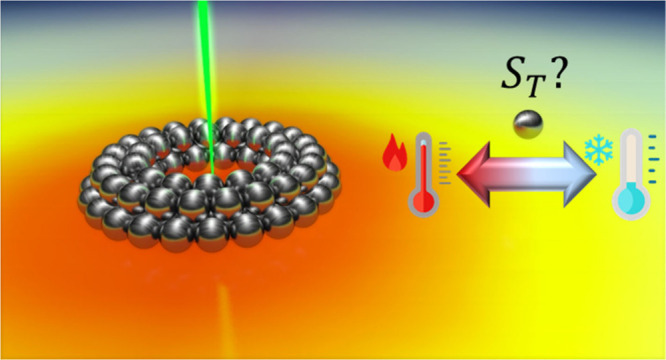

## Background

Thermophoresis, or the Ludwig–Soret effect, describes the motion of colloids within a temperature gradient.^1^ The inhomogeneity arising from the thermal gradient at the particle–solvent interface creates an interfacial tension gradient that, when aligned with the temperature gradient, leads to a pressure disparity within the Debye layer, known as thermo-osmosis, causing it to move toward areas of lower interfacial free energy.^2^ In a dilute suspension in a liquid, the mass flux *J⃗* is defined by

1 $$\mathrm{J⃗}=-D\nabla c-cD_{T}\nabla T$$

where *D* is the mass diffusion or Brownian coefficient, *D*_*T*_ is the thermal diffusion coefficient, and ∇*c* and ∇*T* are the concentration and thermal gradient, respectively. In the steady state, *J⃗* vanishes and eq 1 turns into the following equation

2 $$\nabla c=-c\frac{D_{T}}{D}\nabla T=-cS_{T}\nabla T$$

where *S*_*T*_ is the Soret coefficient, which describes the competition between two opposing coefficients: while *D* tends to homogenize the dispersion, *D*_*T*_ defines an out-of-equilibrium process. Species migrating to colder regions (positive *S*_*T*_) are termed thermophobic, whereas thermophilic species have negative *S*_*T*_. Beyond the classical thermophoretic motion described by the Soret effect, thermal gradients in liquid suspensions can also give rise to a variety of related transport phenomena^3^ (Figure 1). For instance, thermoelectricity, also termed the Seebeck effect, occurs when ions with opposite charges are added to the solution. Due to their differences in *S*_*T*_, they migrate in different directions under a thermal gradient, leading to a spatial separation of charges, resulting in an electric field. Similarly, thermodepletion arises from a temperature-driven concentration gradient beyond the Debye layer (unlike thermo-osmosis), primarily due to the exclusion of nonadsorbing polymers and surfactants from the particle interface.^3^

**Figure 1:**
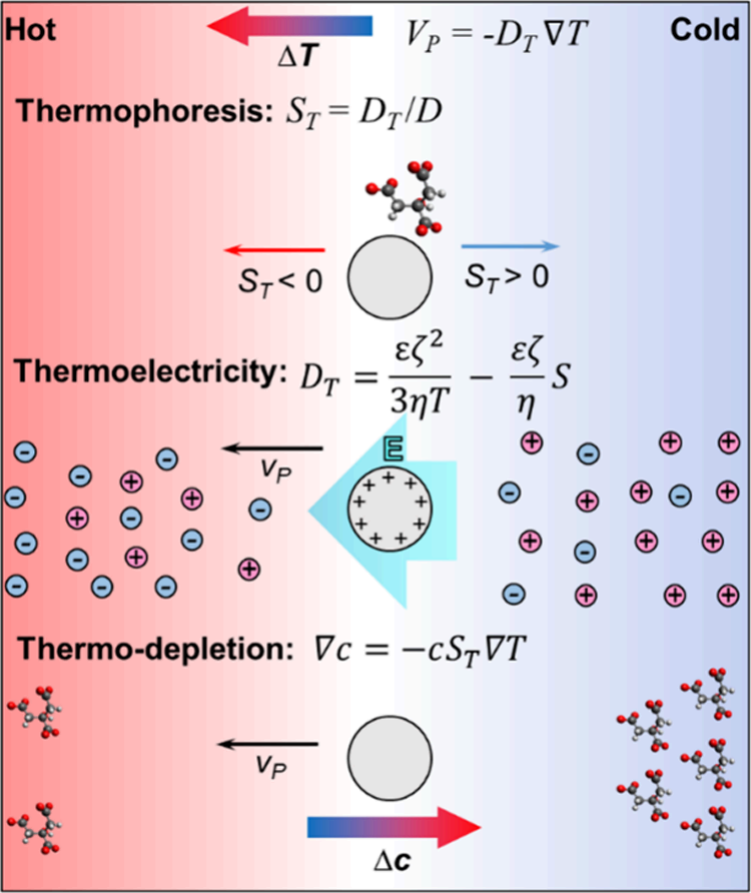
Thermally induced transport phenomena: thermophoresis, thermoelectricity, and thermodepletion.

The coefficient *S*_*T*_ can be divided two additive components: the isotopic *S*_*T*_^*iso*^ and the chemical contribution *S*_*T*_^0^ (eq 3).^4^

3 $$S_{T}=S_{T}^{iso}+S_{T}^{0}$$

The isotopic term, also referred to as the kinetic term, accounts for properties independent of particle–particle interactions, such as mass, moment of inertia, and, as recently reported, mass dipole.^5^ This concept originates from early experiments comparing binary mixtures of isotopes—species with identical chemical interactions but differing in mass—where the variations observed in *S*_*T*_ could be attributed solely to differences in mass and moment of inertia.^6^ Over time, the definition of the isotopic contribution has changed to represent a fundamental property of any mixture where differences in mass and moment of inertia influence thermal diffusion. In contrast, the chemical contribution (*S*_*T*_^0^) encompasses interparticle forces, such as steric and dispersion forces as well as dipolar forces and hydrogen bonding, that become relevant in polar and associating systems. Additionally, in charged systems, electrostatic interactions dominate, making *S*_*T*_ a function of Debye length, where the ionic strength and particle charge strongly influence thermophoretic behavior.

## Current State of Understanding of the Thermophoresis of Colloids

Experimental and computational studies have explored the thermophoresis of surfactants, electrolytes, polymers, micro- and nanosized particles, and biomolecules, shedding light on the influence of size, charge, solvent properties, and other parameters (Figure 2).

**Figure 2:**
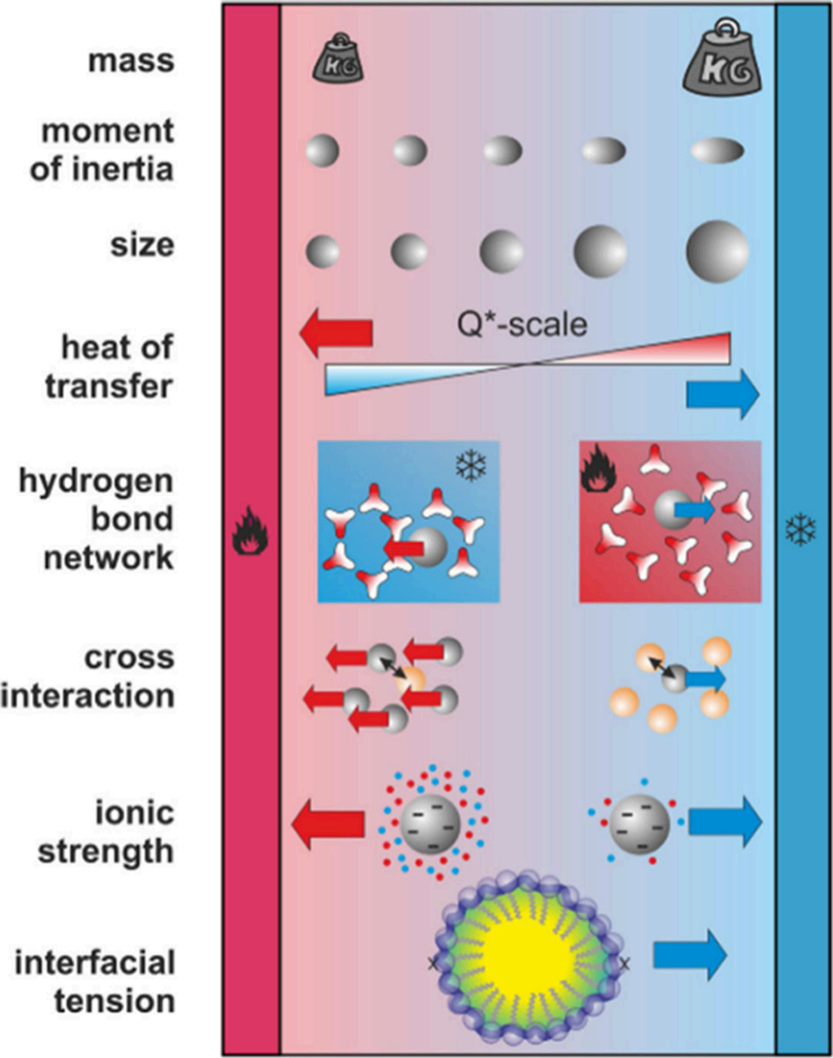
Physicochemical factors influencing the migration of the species under thermal gradients. Reproduced from ref 7, available under a CC-BY 3.0 license.

Polymers have been extensively studied in the context of thermophoresis over the last few decades. One of the most significant findings was the independence of *D*_*T*_ with the degree of polymerization in dilute concentrations.^7^ Interestingly, for polystyrene (PS) in toluene in the concentrated regime (wt % > 50) there is a decay of *D*_*T*_ with increasing concentration that is more pronounced with larger masses, related to its glass-transition temperature.^8^ Several sign changes of *S*_*T*_ induced by changes in solvent composition have also been reported, highlighting the sensitivity of thermophoresis to the colloidal environment. For instance, poly­(vinyl alcohol) (PVA) and polyethylene glycol (PEG) showed a sign inversion from thermophobic to thermophilic in poor solvent (i.e., water and ethanol, respectively) related to hydrogen-bond formation and how this affects their solubility.^9^ While many aqueous systems have shown significant changes with temperature and concentration, this is less common in organic systems. For example, poly *N*-isopropylacrylamide (pNIPAAm) in ethanol exhibits a change from thermophobic to thermophilic upon increasing the temperature above ∼31 °C due to the coil–globule structural transition.^10^

In the case of block copolymers, Pluronic F127 has been reported to have a double sign change along the micellization temperature range: below the critical micelle temperature (cmt), species migrate to the cold side (*S*_*T*_ > 0) while right above it (i.e., single polymer chains and micelles coexisting), species migrate to the hot side (*S*_*T*_ < 0), which may be related to the predominance of hydrophobic interactions in that region.^11^ This sign inversion has not been reported for other self-associating systems such as sodium dodecyl sulfate (SDS) where *S*_*T*_ remains positive along the whole concentration range (below and above the critical micelle concentration, cmc).^12^ These inconsistencies for molecules and polymers, which are relatively well-defined systems, are magnified considerably in the case of colloidal particles in which size, density, shape, and surface chemistry can drastically differ.

PS and silica particles have been the most studied colloidal particles. Factors influencing the *S*_*T*_ of charged spherical particles in aqueous solutions have been studied, highlighting the role of particle size, surface chemistry, or solvent interactions. The influence of the particle size on the magnitude of *S*_*T*_ has not yet been conclusively addressed to date. Experimental results are sometimes contradictory, depending on the theoretical framework used. While some models predict no significant dependence between particle size and *S*_*T*_ using kinetic theory, others suggest a linear correlation when considering thermophoresis by irreversible thermodynamic or hydrodynamic theories. For instance, Braibanti et al.^13^ found no significant influence of particle diameter (ranging from 22 to 506 nm) on the thermophobic migration of PS particles in the temperature range studied (5 °C ≤ *T* ≤ 45 °C). However, Zhou et al. found a sign change of *D*_*T*_ from positive to negative (thermophobic to thermophilic) when increasing the PS particle size from 0.74 to 1 μm.^14^

Several studies have reported the influence of particle surface charge,^15^ pH,^16^ or salt composition in aqueous environments.^17^ Beyond aqueous systems, Ning et al. reported a sign change of *S*_*T*_ from thermophilic to thermophobic of octadecyl-coated silica particles in toluene with increasing temperature (i.e., improving solubility), with the transition temperature increasing at higher concentrations.^18^ This aligns with the previously discussed behavior in polymers, where better solvation corresponds to increased thermophobicity. Pu et al. studied the thermophoresis of silica particles in nonionic and ionic surfactant solutions, finding that the main driving force with nonionic surfactants is the dissociation of silanol functional groups at the particle surface.^19^ On the other hand, in ionic surfactant solutions, thermophoresis of silica particles is predominantly driven by the adsorption of ionic surfactants onto the silica particle surface.

The thermal conductivity of particles plays a crucial role in their thermophoretic behavior, as it directly influences the local temperature gradients at the particle–fluid interface. High thermal conductivity materials, such as gold or silver, tend to homogenize temperature differences across their surfaces, thereby limiting their directed motion and reducing their trapping stiffness compared to materials such as polystyrene and silica.^20^ Particles near surfaces are also influenced by thermo-osmotic slip flows, which arise from interfacial temperature gradients at solid–liquid interfaces and can significantly influence the effective trapping potential by either enhancing or hindering colloidal confinement.^21^ Characterization of these flows has shown that they can directly impact particle trajectories and stability within optical traps, suggesting that they should be considered when designing optothermal trapping and assembly strategies.^22^

Colloid shape can also strongly impact thermophoresis, as water molecules polarize in the direction of the gradient, a phenomenon called thermal orientation.^23^ This effect is particularly relevant for anisotropic particles, either in mass or shape, as the degree of orientation depends on the temperature gradient applied and consequently affects the magnitude of *S*_*T*_.^24,25^ Gittus et al. studied the influence of mass distribution anisotropy on *S*_*T*_, showing that the larger the asymmetry, the more pronounced the decrease in *S*_*T*_, meaning that the thermophoretic force affecting the colloid is significantly reduced by ∼20% for the largest mass dipole (*d* ≈ 4).^26^ These findings clash with the trend observed in spherical colloidal particles, which typically exhibit an increase in *S*_*T*_ with mass.^27^ The thermophoresis of anisotropic particles has been studied in the context of their electrostatic interactions,^24^ shape,^28^ and surface functionalities.^24^ Wang et al. studied the influence of PEG grafting onto the rod-shaped *fd*-virus on their thermal diffusion, concluding that polymer grafting significantly influenced *S*_*T*_ at high ionic strength, although this effect diminishes at low ionic strengths, where electrostatic interactions dominate and both systems exhibit similar behavior.^24^

Aside from the influence of shape, charge, and surface chemistry anisotropy of colloids on their thermophoresis, a nonisotropic fluidic environment can also significantly impact thermophoresis. Along these lines, Kołacz et al. studied the thermophoretic motion of silica beads in liquid-crystalline solvents.^29^ They found that the motion of colloids in such nonisotropic fluidic environments led to elastic distortions, where under sufficiently large local temperature gradients the elastic energy led to negative thermophoresis of the dispersed colloids. This negative thermophoresis was found to have a strongly nonlinear dependence on temperature, related to the temperature-dependent Miesowicz viscosity of the liquid-crystalline solvent. In addition to the strong temperature dependence on the effective viscosity in this nonisotropic liquid environment, very large *S*_*T*_ values (>5000) were observed due to elastophoretic effects and the suppression of Brownian diffusion.

Overall, these findings underscore the complexity of thermophoresis in colloidal systems and the necessity of considering multiple interrelated factors when analyzing their thermal diffusion behavior. Such an understanding is critical for advancing the use of thermophoresis for the assembly and patterning of functional devices for applications. Therefore, to extend the applicability of thermophoresis to advance fields such as nanotechnology and microfluidic systems, it is essential to further develop theoretical models and simulations supported by experimental studies. A deeper understanding of the underlying mechanisms will enable more accurate predictions and control of the thermophoretic behavior.

## Measurement and Prediction of *S*_*T*_

While *S*_*T*_ can exhibit predictable trends in certain cases, it remains system-dependent and sensitive to multiple competing factors (Figure 2). Until the early 2000s, the experimental database of thermal coefficients was limited and often inconsistent across different methods. Advances in new experimental approaches are now providing more reliable benchmark values for *S*_*T*_,^30^ but in spite of the different theoretical approaches, there is not a universally accepted theory for predicting the thermal diffusion coefficient *D*_*T*_. Although a lack of broader understanding presents a challenge to the further development of opto-thermophoresis as a patterning technique of diverse colloids, recent work by various groups continues to add to the toolbox of available experimental and theoretical approaches, which can be used to measure or predict the thermophoretic mobility of colloids and molecules in liquids.

There are a number of experimental techniques for measuring *S*_*T*_, such as field-flow fractionation,^17^ optical beam deflection,^12,13^ fluorescence microscopy,^31^ thermal lensing,^11^ and thermogravimetric columns, all of which are limited to binary systems except for the last. More recently, the techniques of thermal diffusion forced Rayleigh scattering (TDFRS) and diffusion in microfluidic cells have shown promise for measuring thermophoresis in a variety of different systems. TDFRS generates a heterodyne signal which is proportional to the refractive index and when normalized to the thermal signal is directly proportional to *S*_*T*_, and the use of an infrared probe beam is optimal for aqueous solutions.^15^ Microfluidic cells designed with a nonisothermal temperature field have enabled a reliable measurement of *S*_*T*_ and an understanding of the transition between diffusion-dominant and convection-dominant fields.^30^ However, discrepancies can arise due to variations in the experimental system design. For example, in fluidic cells that are too large, natural convection may become significant, whereas in excessively small cells thermo-osmotic effects near the substrate can introduce unwanted noise, complicating data interpretation.

Analytical and theoretical approaches have shown promise for understanding and even predicting *S*_*T*_. Models based on nonequilibrium thermodynamics theory seem to show the best agreement with experimental data,^32^ which relate *D*_*T*_ to certain thermodynamic properties such as heats of transfer. However, this energetic unit is challenging to measure experimentally. To overcome this limitation, alternative approaches within the framework of irreversible thermodynamics have been suggested, either eliminating the need for the direct measurement of such properties or replacing them with other quantities, such as the partial molar activation energy of viscous flow.^33^ Nevertheless, these approaches often rely on broad assumptions, such as considering the activation energy of a component in the mixture to be the same as that of its pure phase, which may hold for nonassociated mixtures but tends to break down in strongly associated systems like alcohols.

Molecular dynamics (MD) simulations have also recently played an important part in understanding thermophoresis. Roemer et al. carried out nonequilibrium MD simulations to calculate the Soret coefficients of alkali halide aqueous solutions and attain a molecular-level view of the process, with the results supported by TDFRS experiments.^34^ Tan et al. used MD simulations for modeling the thermal behavior of a charged rod, finding that thermodiffusion coefficients increased with the aspect ratio and smoother surfaces.^28^ As the parametrization of nanomaterials becomes commonplace, using MD simulations will become a crucial tool to understand how nanoparticle surface chemistry and interactions with solvent and solutes influence thermophoresis.^35^ Despite the advances in simulations that can give a clear molecular-level view of colloidal interactions, the prediction of Soret coefficients in complex mixtures and conditions remains a challenge due to the limited availability of experimental data.

## Outlook and Potential for Advancing Nanofabrication

Several groups have reported the use of laser-generated thermal gradients, termed opto-thermophoresis, to drive colloidal particles toward the hot^21,36^ or cold region.^37^ This has been shown to be a useful laser-based approach for the trapping of colloids which does not involve the high powers or strong optical gradient forces required for classic optical trapping and is compatible with biologically relevant colloids such as vesicles and cells.^38,39^ While opto-thermophoresis has shown promise for the manipulation and trapping of colloids, its potential for directed assembly and printing on solid substrates remains relatively unexplored.

Achieving printing of trapped colloids on solid substrates has so far presented a challenge, which was first overcome by UV-induced polymer cross-linking to freeze the trapped assemblies in place (Figure 3a,b).^40,41^ Only recently have breakthroughs demonstrated the feasibility of using thermophoresis for single-step printing of plasmonic metal nanoparticles via heat-mediated interactions between a dispersed polymer and the substrate interface (Figure 3c,d).^42,43^ However, fundamental challenges persist, including a limited understanding of how interparticle or particle–substrate interactions influence the process, the role of particle morphology, and a limited pool of different nanoparticle compositions which have been studied thus far.

**Figure 3:**
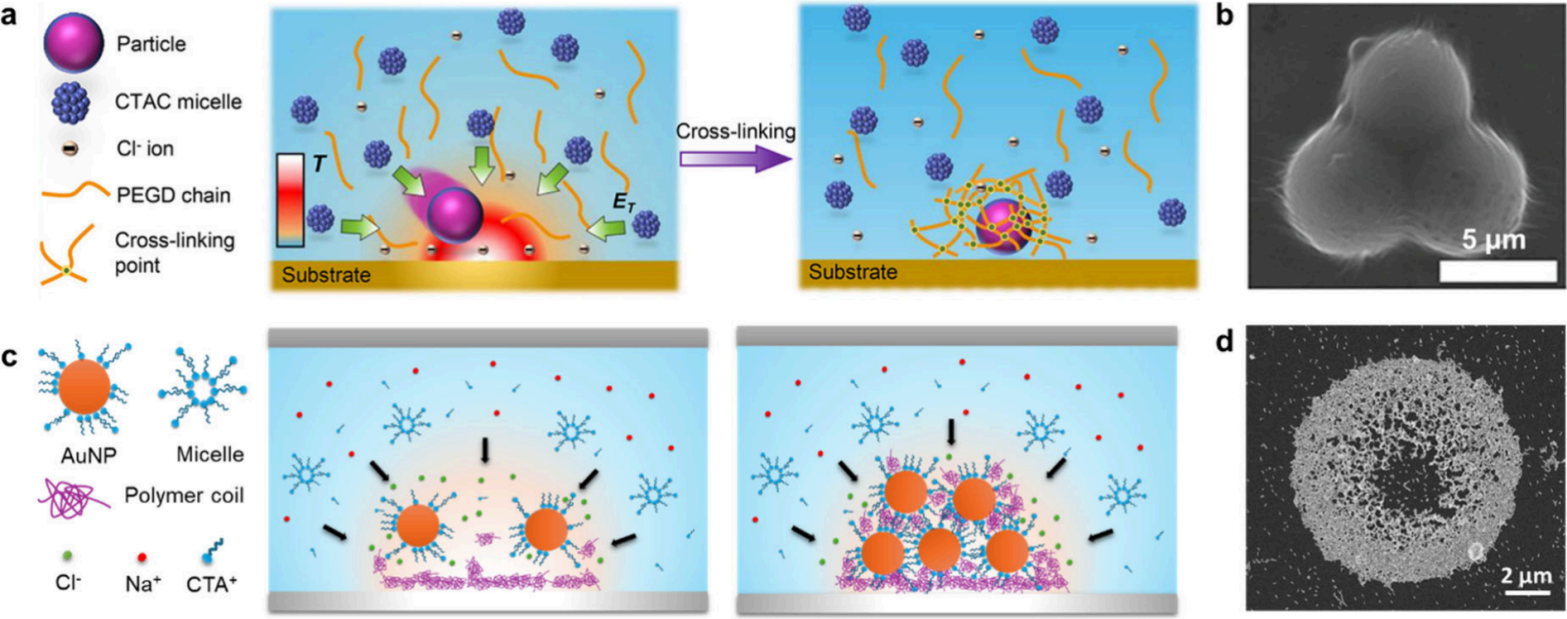
Optothermophoretic printing of colloidal particles on solid substrates. (a) Trapping of a particle induced by a thermoelectric field (left) and immobilization of the particle on the substrate via UV-induced polymer cross-linking (right). (c) Thermophoretic migration of gold nanoparticles and polymer coils to the hot region (i.e., laser spot (left) and adhesion to a glass surface aided by the polymer polyvinylpyrollidone) leading to the assembly and adhesion to the substrate. (b, d) Scanning electron microscopy images of the corresponding printed patterns from a and c, respectively. (a, b) Reprinted with permission from the American Chemical Society.^40^ (c) Reproduced from ref 43, available under a CC-BY-NC 3.0 license. (d) Reproduced from ref 44, available under a CC-BY-4.0 license.

With the current understanding of thermophoresis, developing general strategies that allow the printing of different sizes/shapes/compositions of colloidal particles or even molecules in different colloidal environments is a tough challenge. In this context, recent developments have utilized both experiment and theory to better understand the thermophoretic motion of certain molecules, polymers, and colloidal particles. The slow yet steady progress in research on the thermophoresis of colloids has revealed that, despite the limited number of systems studied (e.g., PEG and PS colloidal particles), the mechanistic understanding and theoretical and experimental approaches have improved to the point where the potential for a paradigm shift in predictive capabilities may be on the horizon. This is an area which could greatly benefit from the ongoing improvements in machine learning for predicting many-body potentials in colloid–polymer mixtures^44^ and the outcomes of colloidal assembly,^45^ which show great promise for advancing this nascent field. The further improvement and refinement of these approaches and their application to a wide range of species from colloidal particles to small molecules will greatly enrich the understanding of thermophoresis in diverse systems and enable more easily generalized strategies for the trapping, sorting, selective concentration, and printing of colloidal species at interfaces.
